# Studies on floral nectary, tepals’ structure, and gynostemium morphology of *Epipactis palustris* (L.) Crantz (*Orchidaceae*)

**DOI:** 10.1007/s00709-014-0668-2

**Published:** 2014-07-29

**Authors:** Agnieszka K. Kowalkowska, Joanna Kostelecka, Jerzy Bohdanowicz, Małgorzata Kapusta, Joanna Rojek

**Affiliations:** Department of Plant Cytology and Embryology, University of Gdańsk, Wita Stwosza 59, 80-308 Gdańsk, Poland

**Keywords:** *Epipactis palustris*, Floral nectar, Self-pollination, SEM, TEM, *Orchidaceae*

## Abstract

The lip of *Epipactis palustris* consists of two movably joined parts: the basal part (hypochile) with central broad isthmus and epichile with callus. The analysis of flowers provides strong evidence to conclude that the whole surface of lip callus and abaxial side of isthmus are secretory. The exudation at first appears on callus, at early stages, later on isthmus. It could be a strategy to prolong the emission of volatile substances and nectar, and this means to prolong luring pollinators. The results from transmission electron microscopy (TEM) support this conclusion. The plastids noted in callus were without starch, whereas the isthmus’ cells contained partly hydrolyzed starch. Some plastids, noted in callus, had polymorphic shapes, which were often related to a starch reduction. During the depletion of starch in callus cells, the number of plastoglobuli within the plastids increased, and also lipid bodies appeared in the cytoplasm whereas, in isthmus cells, proplastids with phytoferritin were noted. The endoplasmic reticulum was in contact with plasmalemma, and the vesicles were fusing with plasmalemma in secretory cells of callus and isthmus, which is a way of granulocrine secretion. The cross-sections of sepals revealed that abaxial epidermis was tomentose, with stomata at the top of substomatal cavities. The pollen grains adhering to the rostellum-viscidium prove previous ecological observations that the rostellum-viscidium is not a barrier preventing self-pollination.

## Introduction

The *Orchidaceae* is the second largest family in angiosperms. The most of representatives reward pollinators, but about one third of species is regarded as deceitful. The most common attractant is nectar (van der Pijl and Dodson 1969; Dressler 1990), gathered in floral and extra-floral nectaries. During floral development, nectar from extra-floral nectaries can be exuded on outer surface of buds or inflorescence (van der Pijl and Dodson 1969), whereas the floral nectaries have various forms: shallow, superficial nectaries on lip surface or nectar spurs produced at the base of labellum or from the fused sepals (van der Pijl and Dodson 1969), or labellar callus (Davies et al. 2005). The nectary in spurs can be formed as an outgrowth from perianth, e.g., from lip base in *Aerangis* Rchb.f. or *Angraecum* Bory and from lateral sepals, lip, and column foot in Spiranthinae (Dressler 1990). In some Laellinae, nectary is embedded in ovary as cuniculus (Dressler 1990). Shallow, superficial nectaries are found in, e.g., *Epipactis atropurpurea* Raf. (Pais 1987), *Listera* R. Br., *Pleurothallis* R. Br., *Stelis* Sw. (Dressler 1990), *Bulbophyllum* Thou. (Endress 1994), *Maxillaria parviflora* (Poepp. & Endl.) Garay (Singer and Koehler 2004), *Bulbophyllum ipanemense* Hoehne, *Bulbophyllum involutum* Borba, Semir & F. Barros, *Bulbophyllum weddellii* (Lindl.) Rchb.f. (Teixeira et al. 2004), and *Bulbophyllum wendlandianum* (Kraenzl.) Dammer (Kowalkowska et al. 2014).

In the genus *Epipactis*, the labellum offers superficial nectar, which attracts, in *Epipactis palustris*, diverse spectrum of visitors, mostly nectar feeders, such as flies, honeybees, and digger wasps (van der Pijl and Dodson 1969; Nilsson 1978; Jakubska-Busse and Kadej 2011). In Polish and Czech populations of *E. palustris*, *Diptera* (*Empis* sp., *Episyrphus* sp.) and Hymenoptera (*Apis mellifera*, *Bombus lapidarius*, *Bombus lucorum*) were noted as the pollinators (Jakubska-Busse and Kadej 2011). The main attractants responsible for enticing insects in Marsh Helleborine’s nectar are as follows: nonanal (pelargonaldehyde), decanal, eicosanol, and its derivatives. The scent composition emitted by flowers, containing strong aromatic compounds as eugenol and vanillin, might be the most crucial in initially attracting *Diptera* pollinators (Jakubska-Busse and Kadej 2011).

The gynostemium morphology in flowers of *Epipactis* species displays the allogamous and autogamous characters (Bonatti et al. 2006). The gynostemium usually has a well-developed, simple type of rostellum. The rostellum is the swollen apical part from the median stigmatic lobe. The tip of rostellum produces adhesive substance, forming a viscidium (Schick 1989). The occurrence of the rostellum-viscidium is an attribute generally associated with cross-pollination, whereas its lack, accompanied by friable pollinia and stigmatic hypersecretion, is related to self-pollination (Robatsch 1983). In *E. palustris*, the median stigmatic lobe is wider than the lateral ones, and large, prominent, and almost globular rostellum is situated above a broad squarish stigma, like in *Epipactis helleborine* (Bonatti et al. 2006). Tałałaj and Brzosko (2008) noted that geitonogamy is observed, but dominant way of pollination is allogamy. On the other hand, Jakubska-Busse and Kadej (2008, 2011) claimed that the long list of insects visiting the flowers and the nectar rich in aromatic compounds suggests that self-pollination in *E. palustris* is not related to its floral structure and is not caused by the lack of potential pollinators or a poor luring strategy, but rather geitonogamy is a result of pollinators’ biology (mostly Vespidae and Apidae).

Although pollination mechanism of *E. palustris* is quite well studied, flower structure of this species is weakly examined, and no ultrastructural studies were done, and also, the information about nectary is inconsistent (Nilsson 1978; Brantjes 1981; Szlachetko and Skakuj 1996; van der Cingel 1995; Jakubska and Kadej 2006, 2008). In this paper, we want to provide micromorphological, histochemical, and ultrastructural studies on lip nectary and tepals’ structure of *E. palustris*. We also give some details indicating the high possibility of self-pollination occurrence.

## Materials and methods

**Fig. 1:**
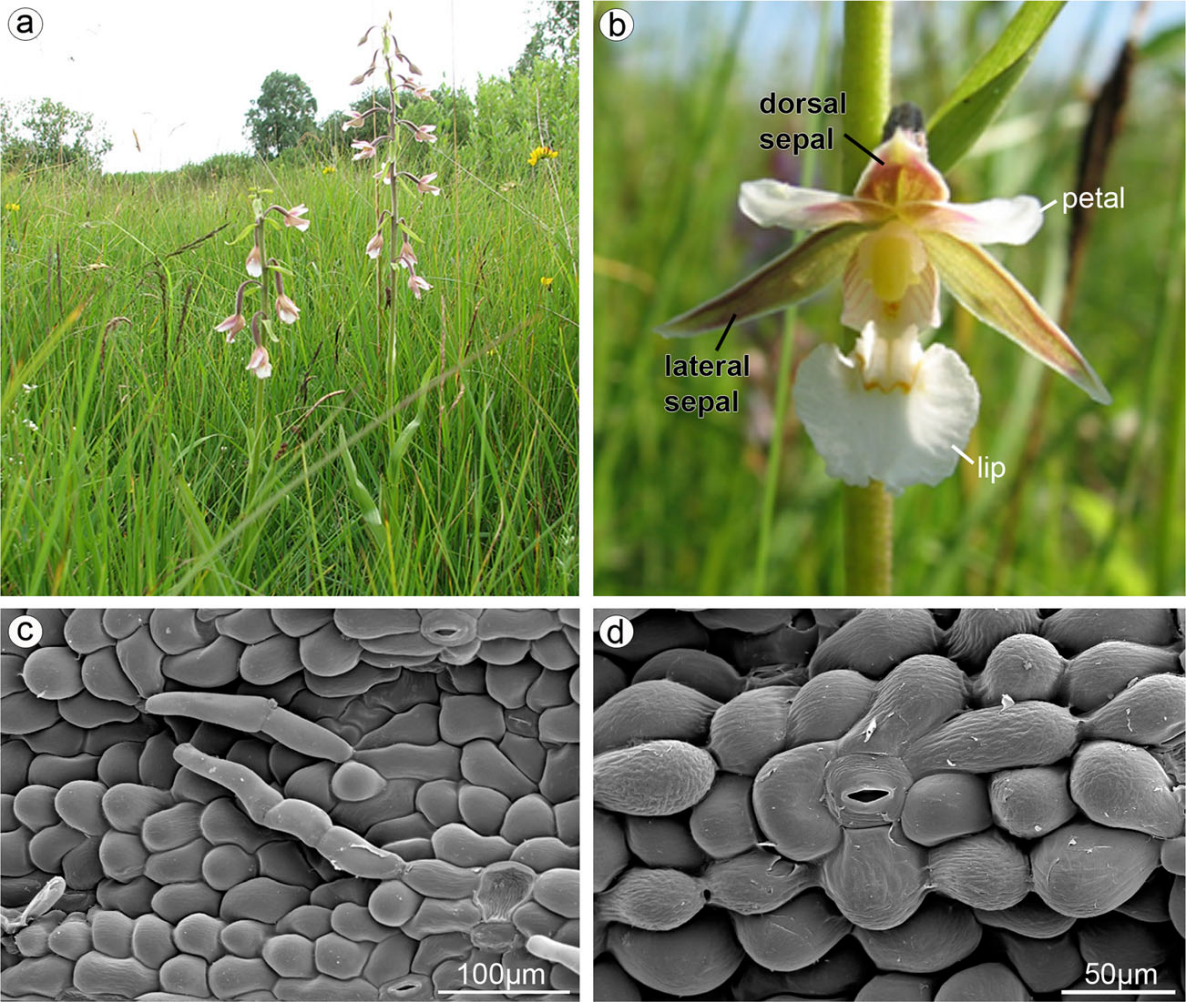
*Epipactis palustris*. **a** Plant with a loose raceme. **b** The outer whorl consisted of dorsal sepal and two lateral sepals, and the inner whorl consisted of two petals and lip. **c** The abaxial side of dorsal sepal, whole surface covered by three- or four-cellular trichomes, numerous stomata present (SEM). **d** Anomocytic stomata on the top of abaxial ridge on the abaxial side of dorsal sepal (SEM)

Flowers of *E. palustris* were collected from plants growing in Northern Poland (Nadleśnictwo Wejherowo, Leśnictwo Orle, oddz. 47, and Mechowiska Sulęczyńskie) (Fig. 1a, b). Fresh flowers were observed under a Nikon SMZ1500 stereomicroscope. Pieces of tepals and labellum tissue were fixed in 2.5 % glutaraldehyde (GA) in 0.05 M cacodylate buffer (pH = 7.0). The material for light microscopy (LM) was rinsed with cacodylate buffer and then dehydrated. The dehydrated material was embedded in epoxy resin (Spurr 1969) and methyl methacrylate-based resin (Technovit 7100, Heraeus Kulzer GmbH). Sections (1–5 μm thick) were cut with glass knives and mounted on glass slides. For LM, the material was stained with 0.05 % toluidine blue O (TBO) for 1 min at 60 °C on a hot plate (Feder and O’Brien 1968; Ruzin 1999). Aniline blue black (ABB, C.I. 20470) was used for detection of water-insoluble proteins (Jensen 1962). The periodic acid-Schiff (PAS) reaction was used to identify the presence of water-insoluble polysaccharides (Jensen 1962) and Sudan Black B (SBB) for lipid localization (Bronner 1975). For scanning electron microscopy (SEM), after dehydration in an ethanol series, the samples were dried by the critical point method using liquid CO_2_, coated with gold and observed in a Philips XL-30. For transmission electron microscopy (TEM), the floral material was fixed in 2.5 % GA in 0.05 M cacodylate buffer (pH 7.0). The material was postfixed overnight in 1 % OsO4 in cacodylate buffer in a refrigerator and then rinsed in the buffer. After 1 h in 1 % uranyl acetate in distilled water, the material was dehydrated with acetone and embedded in Spurr’s resin. Ultrathin sections were cut on a Sorvall MT 2B ultramicrotome with a diamond knife and contrasted with uranyl acetate and lead citrate. The sections were examined in a Philips CM 100 transmission electron microscope. Samples were prepared in accordance with procedures described elsewhere (Kowalkowska and Margońska 2009; Kowalkowska et al 2010, 2012).

Lips were fixed in acetic alcohol (1:3 glacial acetic acid/96 % ethanol); after 1–2 weeks, the material was placed in 70 % ethanol. Permanent slides were prepared using the standard paraffin method. Microtome sections (10 μm thick) were stained with Ehrlich’s hematoxylin combined with alcian blue (Ruzin 1999) and mounted in DPX (Fluka).

The preparations (TBO, ABB, PAS, SBB, paraffin method) were examined and photographed with a Nikon Eclipse E800 light microscope equipped with a Nikon DS-5Mc camera and analyzed with Lucia Image software.

## Results

**Fig. 2:**
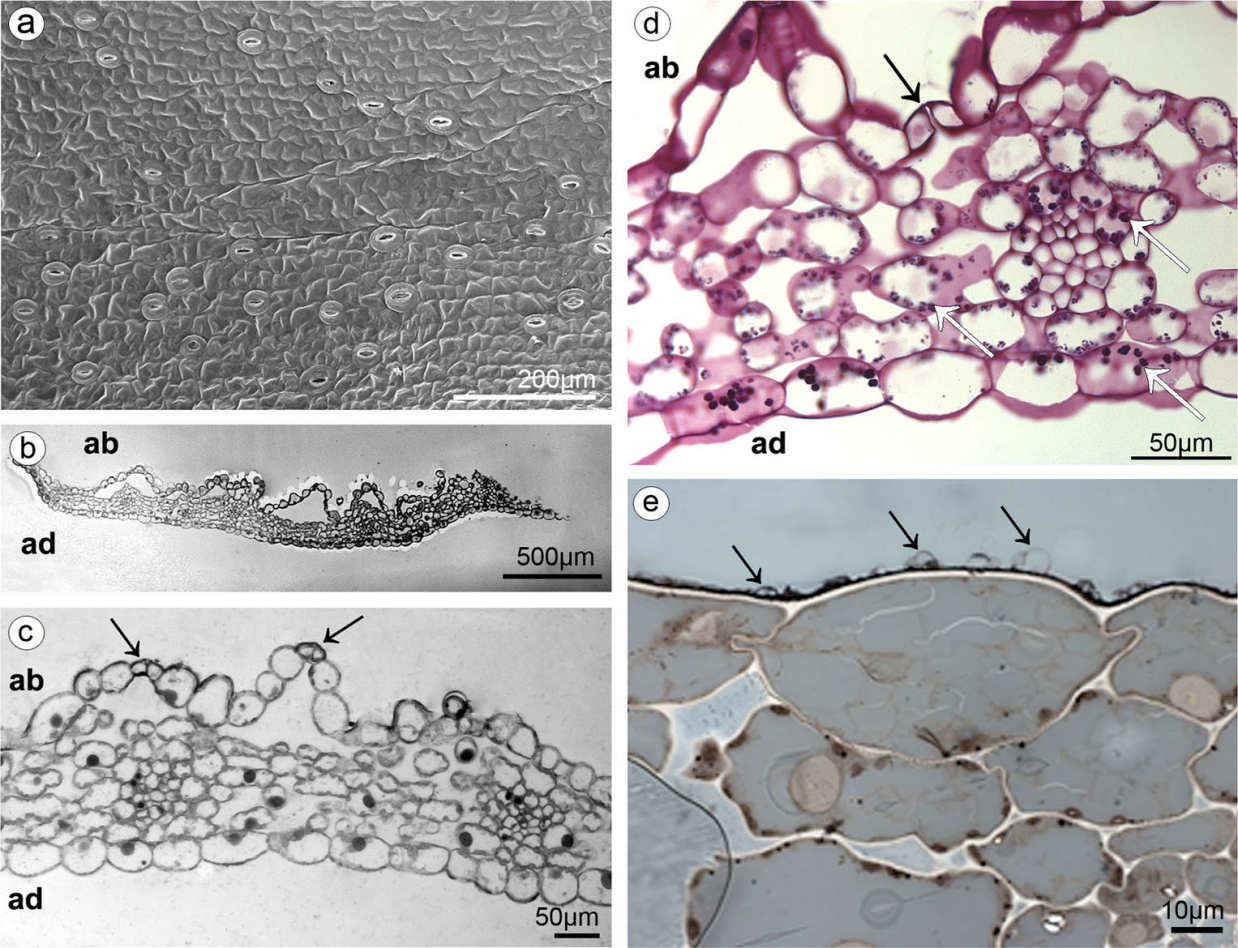
**a** The adaxial glabrous surface of dorsal sepal with numerous anomocytic stomata close to the apex (SEM). **b** Cross-section of dorsal sepal with high elevations on abaxial side (TBO). **c** Magnification of **b**, with stomata at the top of elevations—substomatal cavities (*arrows*) (ABB). **d** Magnification of **b**, stomata also present in epidermis between elevations (*black arrow*) and starch grains noted (*white arrows*) (PAS). **e** The adaxial side with numerous lipid bodies and blisters on the cells (*arrows*) (SBB) (*ad* adaxial (inner) surface, *ab* abaxial (outer) surface)

**Fig. 3:**
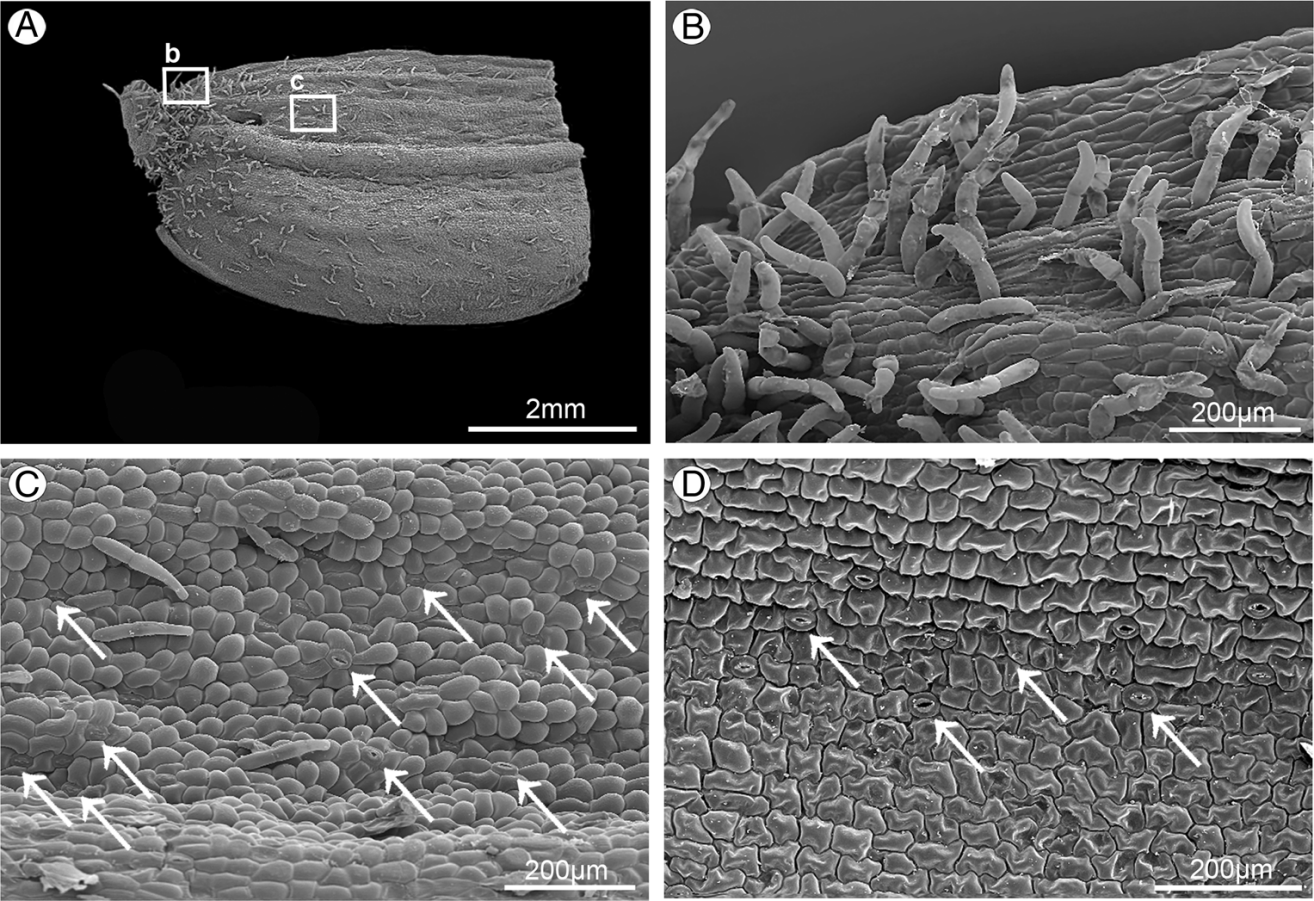
Surface of lateral sepal (SEM). **A** The base of abaxial side with central keel (*rectangle b - part magnified on* ***b***, *rectangle c - part magnified on* ***c***). **B** Magnification of **A**, the base part, with three-cellular trichomes. **C** Magnification of **A**, trichomes and numerous anomocytic stomata (*arrows*). **D** Glabrous adaxial side with numerous anomocytic stomata (*arrows*)

**Fig. 4:**
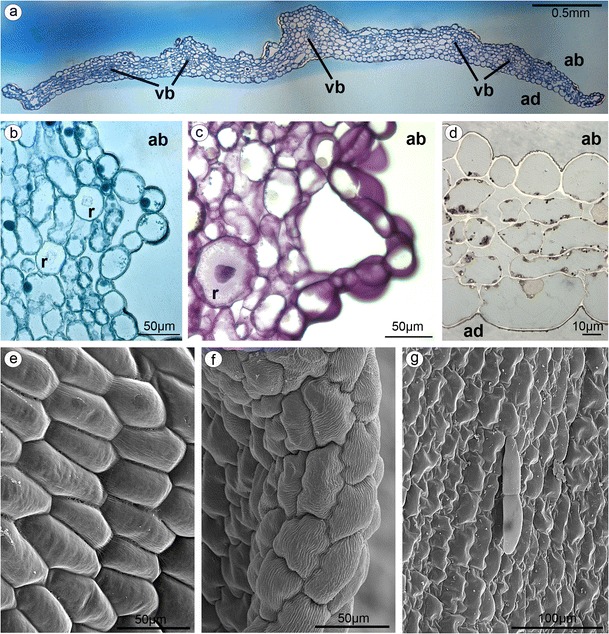
**a** Cross-section of lateral sepal with collateral vascular bundles (*vb*) in parenchyma (*ad* adaxial (inner) surface, *ab* abaxial (outer) surface, TBO). **b** Lateral sepal—staining for proteins (ABB), idioblasts with raphides (*r*) present in parenchyma. **c** Lateral sepal—staining for insoluble polysaccharides (PAS), raphides (*r*). **d** Lateral sepal—staining for lipids (SBB), numerous lipid bodies in cells. **e** The abaxial surface of petal built by polygonal cells with straight walls (SEM). **f** The apex and inner (adaxial) surface of petal with polygonal cells with the U-shaped walls (SEM). **g** The three-cellular trichomes (one basal cell and two protruding cells) rarely present on adaxial petal surface (SEM)

The whitish, widely open, spurless flowers were borne in a loose raceme (Fig. 1a, b). The dorsal and lateral sepals were parts of outer whorl. They were broadly and distinctly abaxially (dorsally) keeled (Fig. 3A). The abaxial (outer) side of sepals, especially near the base and along the median nerve, was tomentose, built by three- or four-cellular trichomes (Figs. 1c and 3A–C). The basal cell of such trichome was immersed in the epidermis, and the other cells protruded above. The adaxial (inner) surface was glabrous (Figs. 2a and 3D). The numerous anomocytic stomata were present on both surfaces: especially on the top of abaxial ridges (Figs. 1d and 3C) and adaxially, along the midnerve, especially close to the apex (Figs. 2a and 3D). The cross-sections of sepals revealed that single-layered abaxial epidermis was irregularly highly elevated (Figs. 2b, c and 4a–c). These elevations were the substomatal cavities with the stomata at the top of such elevation (Fig. 2c). Numerous anomocytic stomata were also visible between such elevations (Fig. 2d). Stain for insoluble proteins (ABB) did not detect more quantity of proteins inside the cells (Figs. 2c and 4b). In PAS method, starch grains, gathered close to the cell walls, were visible (Figs. 2d and 4c). In the inner epidermis, starch grains were present close to the cell walls, which were adjacent to the parenchyma cells (Fig. 2d). SBB staining revealed numerous lipid bodies in cells (Figs. 2e and 4d), also some blisters on adaxial (inner) surface of dorsal sepal (Fig. 2e). The idioblasts with raphides were present underneath epidermis (Fig. 4b, c).

Petals and lip were parts of inner whorl and were white, purplish at base (Fig. 1b). The abaxial (dorsal, outer) surface of petals were built by polygonal cells with straight walls (Fig. 4e), whereas the cells on the apex and inner (adaxial) surface were polygonal with the U-shaped walls (Fig. 4f, g). The three-cellular trichomes (one basal cell and two protruding cells) were rarely present (Fig. 4g).

**Fig. 5:**
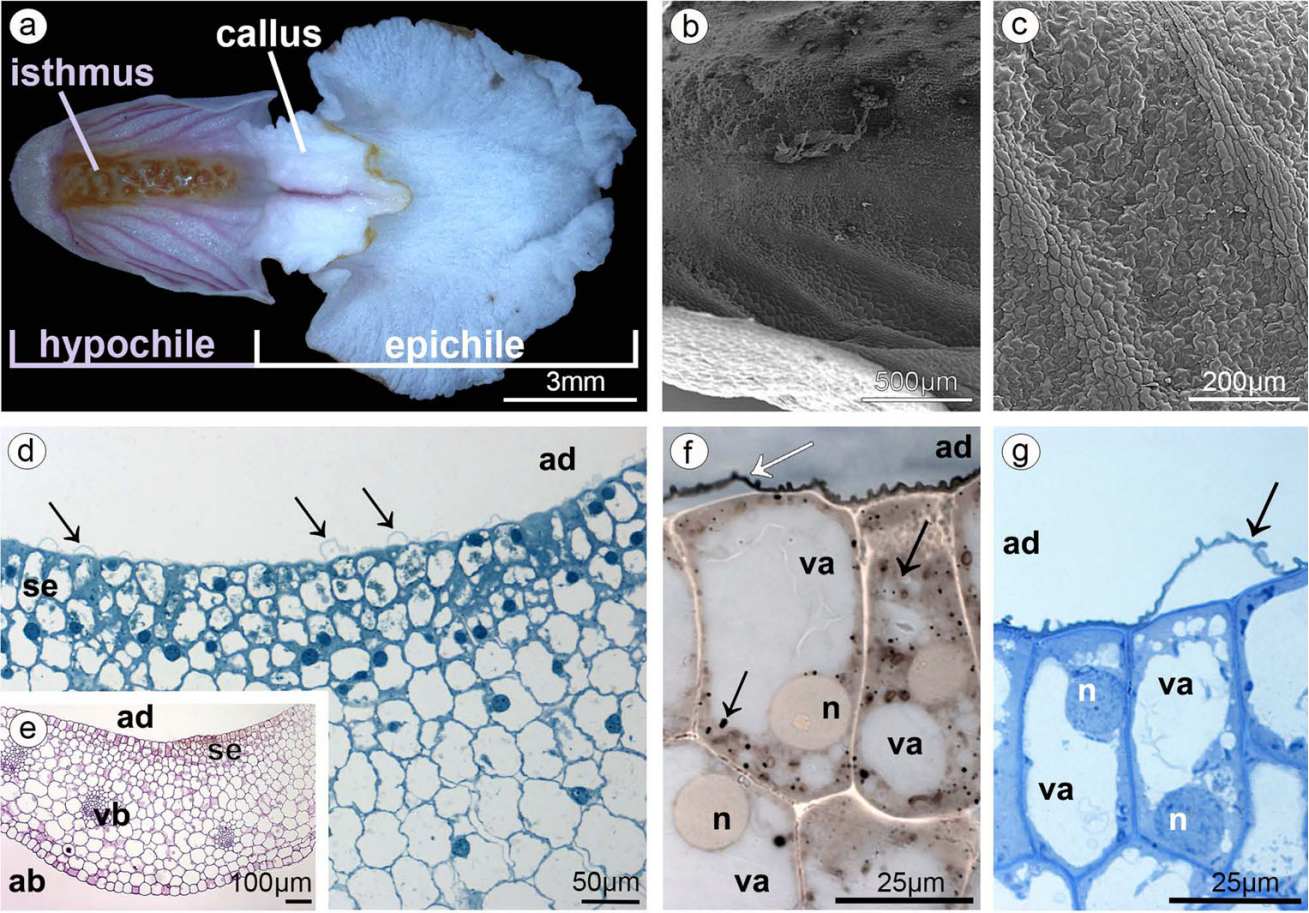
**a** Lip with two movably joined parts: the basal part (hypochile) consisted of concave central broad isthmus with orange-yellow bulges and two triangular lateral lobes, marked with purple parallel veins and white epichile with central callus and strongly undulate margins. **b** The isthmus cells covered by secretory residue. **c** The adaxial (inner) surface of lateral lobes covered by smooth polygonal cells with wrinkled cuticle (SEM). **d** Dense cytoplasm, more intensively stained on proteins in epidermal and few subepidermal cells (*se* secretory epidermis) with distinctly distended cuticle (*arrows*) (*ad* adaxial (inner) surface, ABB). **e** The cross-sections of isthmus with secretory epidermis (*se*), vascular bundles (*vb*) in parenchyma (*ad* adaxial (inner) surface, *ab* abaxial (outer) surface, PAS). **f** Isthmus cells with distended cuticle (*white arrow*) with large vacuoles (*va*) and nucleus (*n*) and numerous lipid bodies (*black arrows*) (SBB). **g** Secretory isthmus epidermis with distended cuticle (*arrow*), cells with large vacuoles (*va*) and nucleus (*n*) (TBO)

**Fig. 6:**
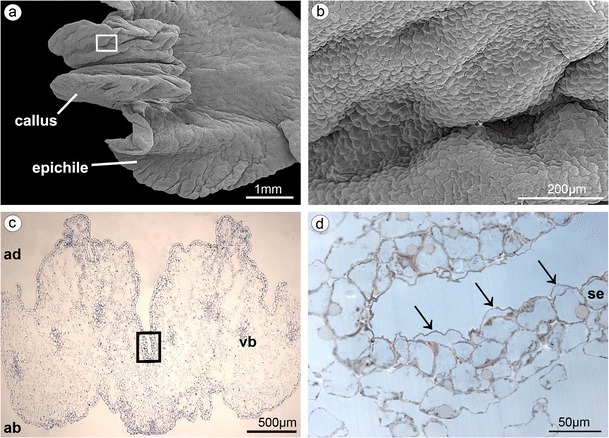
**a** Fleshy callus placed at the base of the epichile (SEM). **b** Magnification of **a**, callus built by polygonal cells with U-shaped cell walls. **c** Cross-section of callus with central groove and collateral vascular bundles in parenchyma (*ad* adaxial (inner) surface, *ab* abaxial (outer) surface, TBO). **d** Central groove of callus with bulges of distended cuticle (*arrows*) on secretory epidermis (*se*) (SBB)

**Fig. 7:**
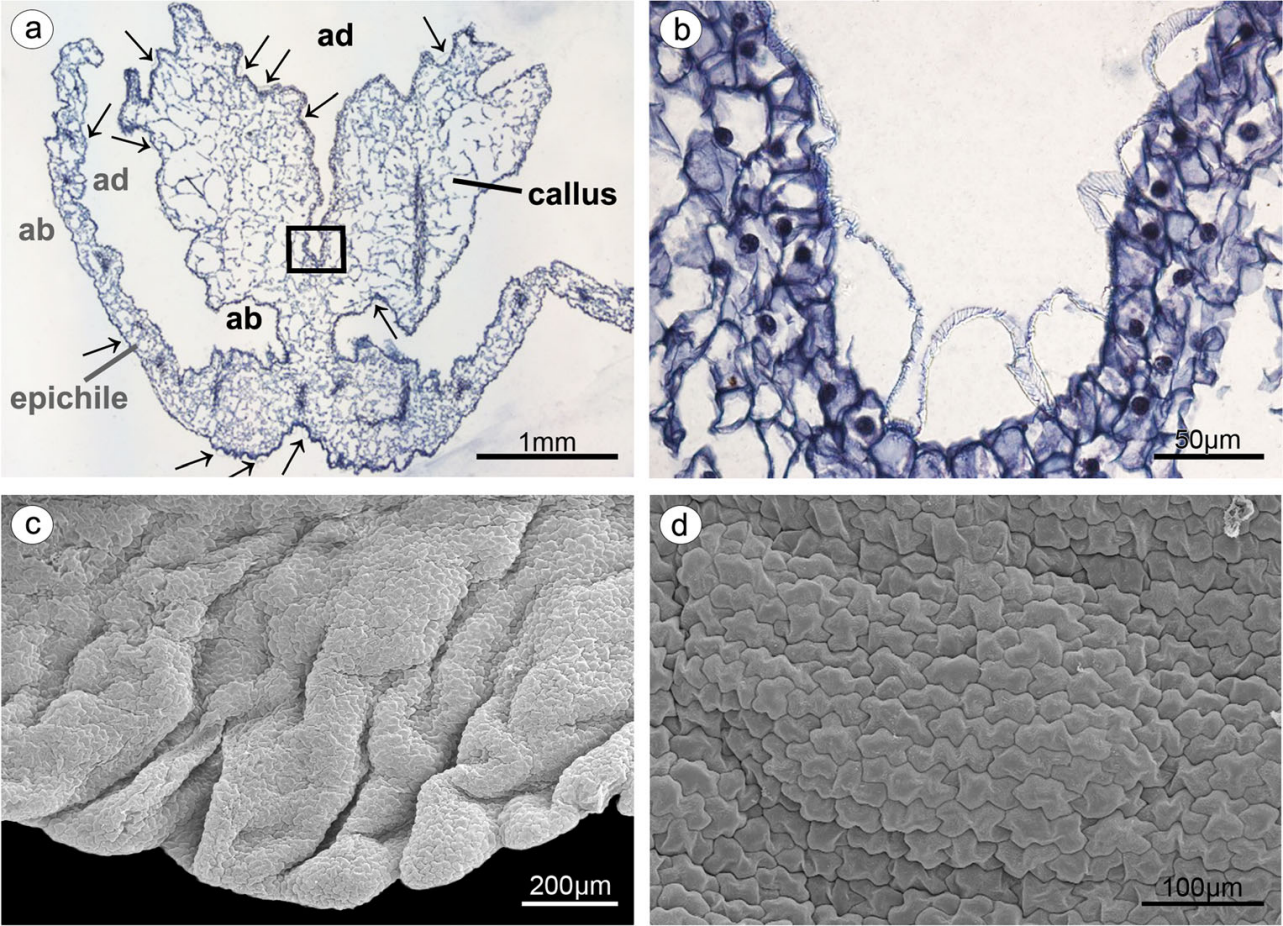
**a** Epichile cross-section of the epichile (tepal and callus) with distended cuticle (*arrows*) on adaxial surface (*ad*) and abaxial surface (*ab*) (paraffin method). **b** Magnification of **a**, central groove with distended cuticle (paraffin method). **c** Strongly undulate margins of epichile (SEM). **d** Magnification of **c**, the epichile surface built by polygonal cells with U-shaped cell walls (SEM)

The lip consisted of two movably joined parts (Fig. 5a). The basal part (hypochile) was whitish and consisted of concave central broad isthmus with orange-yellow bulges and two triangular lateral lobes, marked with purple parallel veins. The isthmus cells of hypochile were covered by secretory residue (Fig. 5b). The adaxial (inner) surface of lateral lobes was covered by smooth polygonal cells with wrinkled cuticle (Fig. 5c). The cross-sections of isthmus (Fig. 5e) revealed that the cytoplasm in epidermal and few subepidermal cells was more dense compared to that in parenchyma cells and more intensively stained on proteins (Fig. 5d). The cuticle was distinctly distended in many places of isthmus, and such areas were not stained on polysaccharides, proteins (Fig. 5d), or lipids (Fig. 5f). The bulges of distended cuticle appeared on the surface upon the border of neighboring cells and distended further on the whole cell surface (compare Figs. 5f, g, 6d, and 7b with 8c, e). Few fine lipid bodies were detected in the epidermal and subepidermal cells (Fig. 5f). The callus (Fig. 6a), placed at the base of the epichile, was fleshy, vivid yellow to orange in front and white with pink color in the middle (Fig. 1b). Its surface was strongly undulated, built by polygonal cells with U-shaped cell walls (Fig. 6a, b). The distended cuticle was observed on the whole callus surface (Fig. 7a), especially in the central groove, Figs. 6c, d and 7b) and also on the abaxial surface of epichile, where callus was located (Fig. 7a). The secretory activity was observed in single-layered epidermis. Underneath the epidermis, parenchyma with large intercellular spaces and collateral vascular bundles was visible (Fig. 7d). PAS method and ABB staining did not reveal more polysaccharides and proteins in callus cells (not illustrated). The epichile, joined to the hypochile by a flexible and elastic hinge, was arcuate, roundish and white (Figs. 1b and 5a). The margins were strongly undulated (Fig. 7c), curved upward near the midlobe apex, built by polygonal cells with U-shaped cell walls, the same as on petals and callus (Fig. 7d, compare with Figs. 4f, g and 6b). There were no noticeable secretions on the epichile surface (Fig. 7d). The vascular bundles in tissues were collateral (Figs. 2c, d, 4a, 5e, and 6c).

**Fig. 8:**
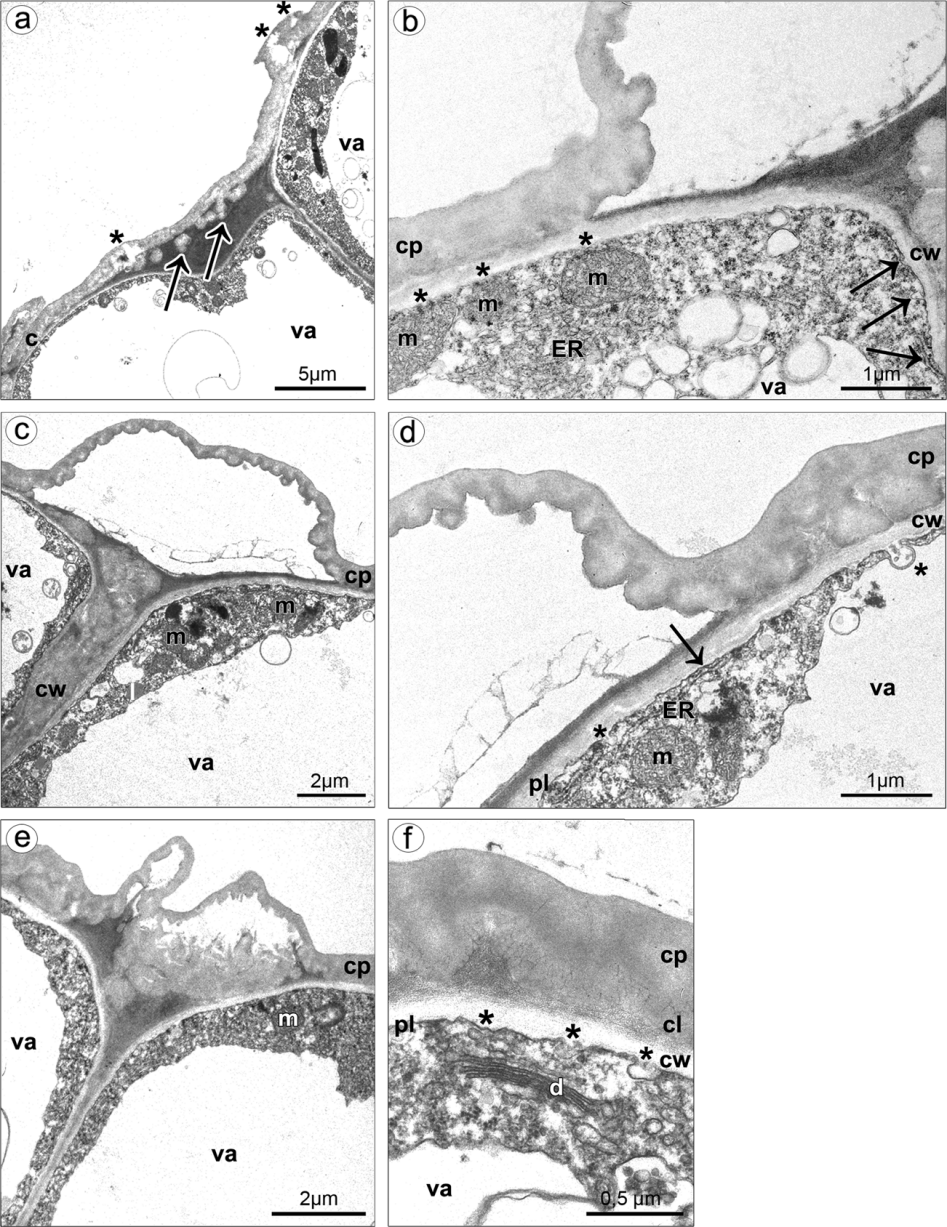
Ultrastructure of callus cells. **a** The swelled and almost ruptured cuticle (*asterisks*) caused by pressure of gathered substances, the globules noted beneath the cuticle (*arrows*). **b** Spherical mitochondria located near the plasmalemma (*asterisks*), profiles of ER in contact with plasmalemma (*arrows*). **c** The bulges of distended cuticle on the surface upon the border of neighboring cells and distended further on the whole cell surface, the cell volume occupied by a central vacuole, parietal cytoplasm. **d** Profiles of ER in contact with plasmalemma (*arrow*), vesicles building into plasmalemma (*asterisks*), the cuticle consisted of cuticle proper and reticulate cuticle layer. **e** The swelled cuticle on the surface upon the border of neighboring cells. **f** Vesicles building into plasmalemma (*asterisks*), dictyosome (*c* cuticle, *cl* cuticle layer, *cp* cuticle proper, *cw* cell wall, *d* dictyosome, *ER* endoplasmic reticulum, *m* mitochondrion, *pl* plasmalemma, *va* vacuole)

**Fig. 9:**
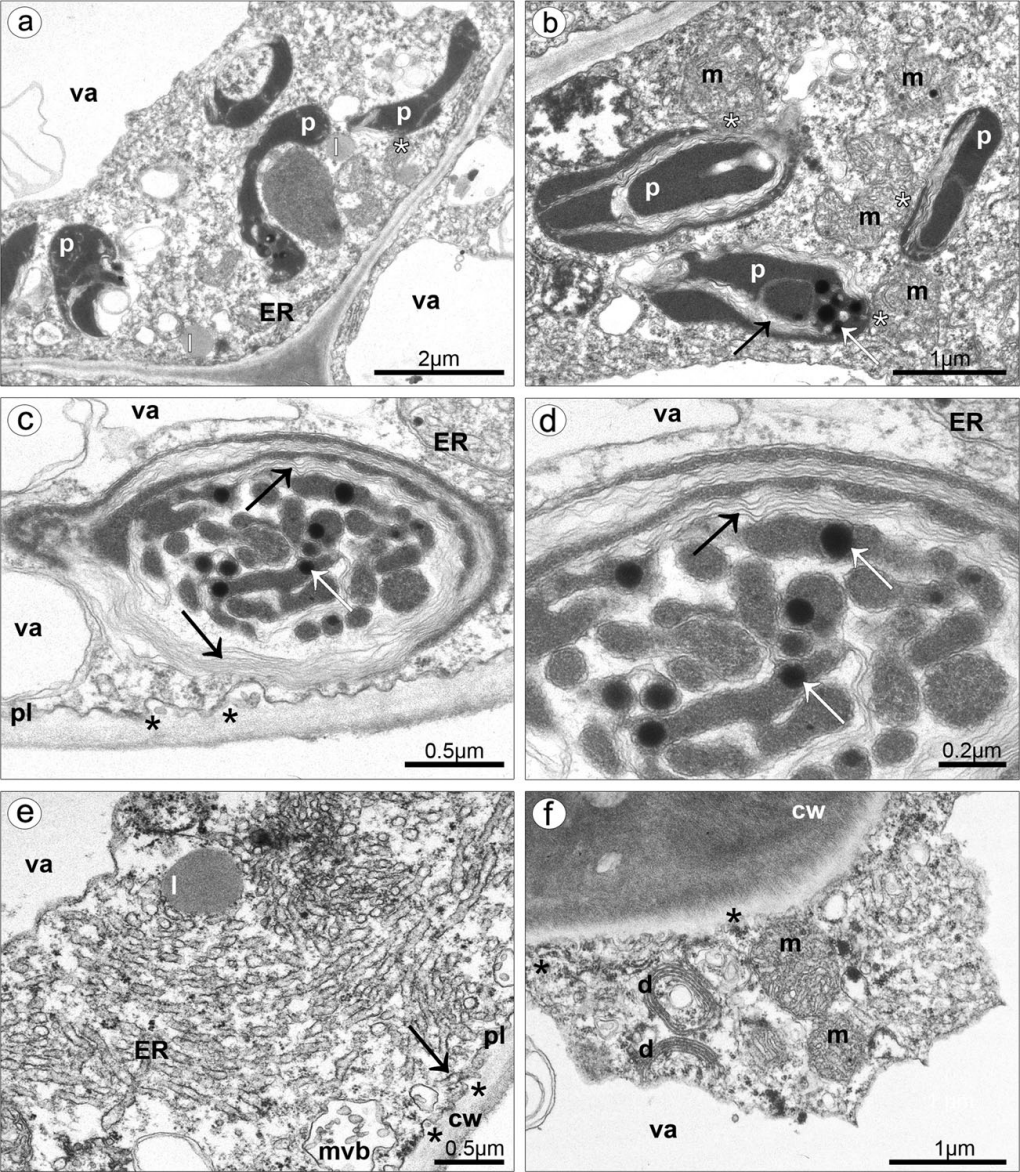
Ultrastructure of callus cells. **a** Oval and cup-like plastids in vicinity of lipid bodies and ER (*asterisk*). **b** Plastids with internal membranes (*black arrow*) and plastoglobuli (*white arrow*) connected with mitochondria (*asterisks*). **c** Plastid matrix with system of internal membranes (*black arrows*) and plastoglobuli (*white arrow*), vesicles building into plasmalemma (*asterisks*). **d** Magnification of **c. e** Lipid bodies connected with the expanded system of ER profiles, also in connection with plasmalemma (*arrow*), multivesicular body close to and vesicles building into the plasmalemma. **f** Profiles of ER close to or being in contact with plasmalemma (*asterisks*), well-developed dictyosomes surrounded vesicles (*cw* cell wall, *d* dictyosome, *ER* endoplasmic reticulum, *l* lipid body, *m* mitochondrion, *mvl* multivesicular body, *pl* plasmalemma, *va* vacuole)

TEM studies of callus cells displayed some typical features of flowers at anthesis. Much of the cell volume was occupied by a central vacuole, and the cytoplasm was visible as parietal layer (Fig. 8a, c, e). The cuticle on outer tangential walls was consisted of cuticle proper and reticulate cuticle layer (Fig. 8d, f). Beneath the cuticle, the globules were noted (Fig. 8a). In case of pressure of gathered substances, the cuticle swelled and ruptured (Fig. 8a–e). The vesicles building into the irregular plasmalemma were sometimes present (Fig. 8d, f). The shape of plastids was polymorphic: oval (Fig. 9b–d) or cup-like (Fig. 9a). They were noted near the cell wall of some cells, in the lower layers of callus parenchyma. System of internal membranes strongly divided the plastids’ matrix (Fig. 9c, d). Plastids contained many plastoglobuli and almost hydrolyzed starch grains. Lipid bodies were sometimes noted (Fig. 9a, e), connected with profiles of ER, which could arrange the expanded system of tubules (Fig. 9e). Profiles of ER were also noted close to or being in contact with plasmalemma (Figs. 8b, d and 9e, f). Free ribosomes were present in cytoplasm (Fig. 8b). Spherical mitochondria were abundant in cytoplasm (Figs. 8b–f and 9b, f), sometimes near plasmalemma (Fig. 8b) and sometimes connected with plastids (Fig. 9b). The dictyosomes were well developed (Fig. 8f) and sometimes surrounded by vesicles (Fig. 9f).

**Fig. 10:**
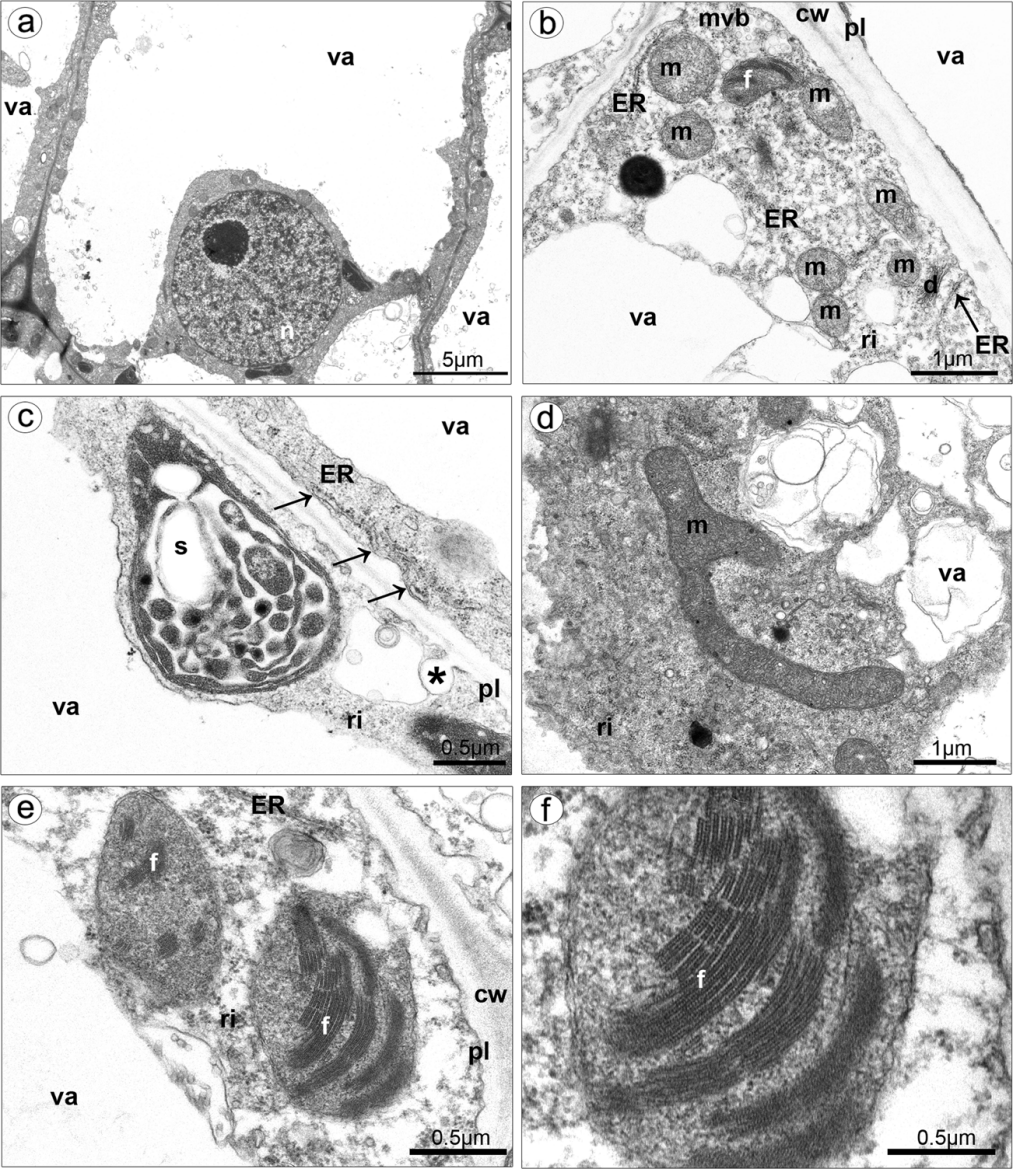
Ultrastructure of isthmus cells. **a** Highly vacuolated cells, with large nucleus. **b** In cytoplasm, presence of numerous mitochondria, plastid with phytoferritin, dictyosome, multivesicular body, free ribosomes, and ER in contact with plasmalemma. **c** Plastid with partially hydrolyzed starch grains, plastoglobuli, and internal membranes, ER in contact with plasmalemma (*arrows*), invagination of plasmalemma. **d** Branched mitochondrion. **e** Proplastids with phytoferritin. **f** Magnification of **e** (*cw* cell wall, *d* dictyosome, *ER* endoplasmic reticulum, *f* phytoferritin, *l* lipid body, *m* mitochondrion, *mvl* multivesicular body, *pl* plasmalemma, *ri* ribosome, *s* starch grain, *va* vacuole)

**Fig. 11:**
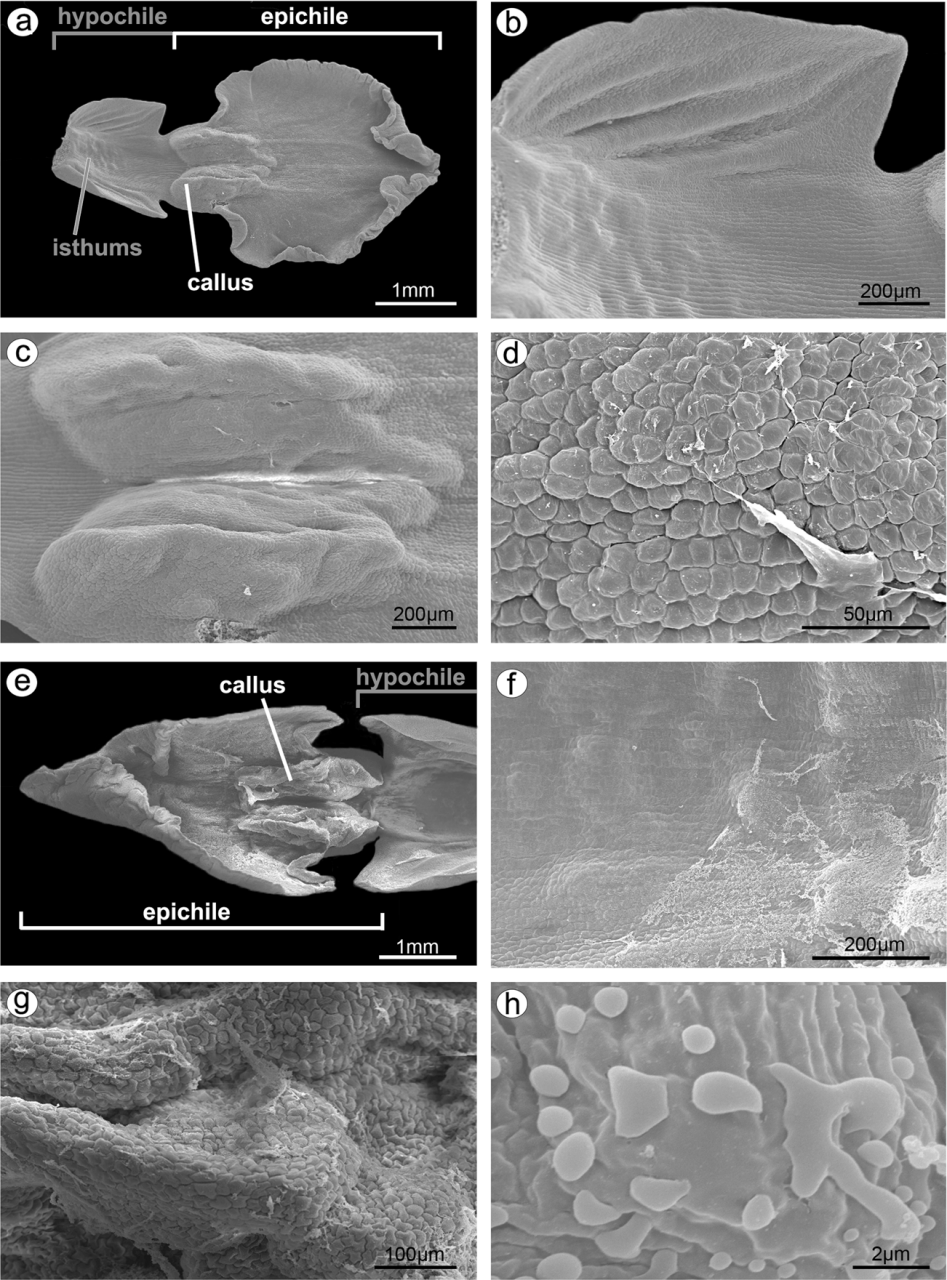
Micromorphology of buds. **a** Lip with callus measuring about 1 mm. **b** Magnification of **a**, no exudation on isthmus cells. **c** Magnification of **a**, exudation noted on callus cells. **d** Magnification of **c**, exudation on callus cells. **e** Lip with callus measuring about 1.8 mm. **f** Exudation visible on isthmus cells. **g** Abundant secretion on callus cells. **h** Magnification of **g**, exudation on callus cells

The isthmus cells were highly vacuolated, with large nucleus (Fig. 10a). The parietal layer of cytoplasm contained profiles of smooth and rough ER, free ribosomes, numerous mitochondria: spherical or with branched shape (Fig. 10b, d). The plastids possessed starch grains, plastoglobuli, and internal membranes (Fig. 10c). There were also visible proplastids with phytoferritin—storage form of Fe (Fig. 10b, e, f). Comparing the different buds’ sizes, it was noticeable that the exudation on lip callus appeared earlier than on isthmus (Fig. 11). In bud with callus measuring about 1 mm (Fig. 11a), the secretion was only noted on callus (Fig. 11c, d), not on isthmus (Fig. 11b). In bud with callus measuring about 1.8 mm (Fig. 11e), the secretion on callus was abundant (Fig. 11g, h), and the exudation on isthmus was also noted, but not in so copious amount (Fig. 11f).

**Fig. 12:**
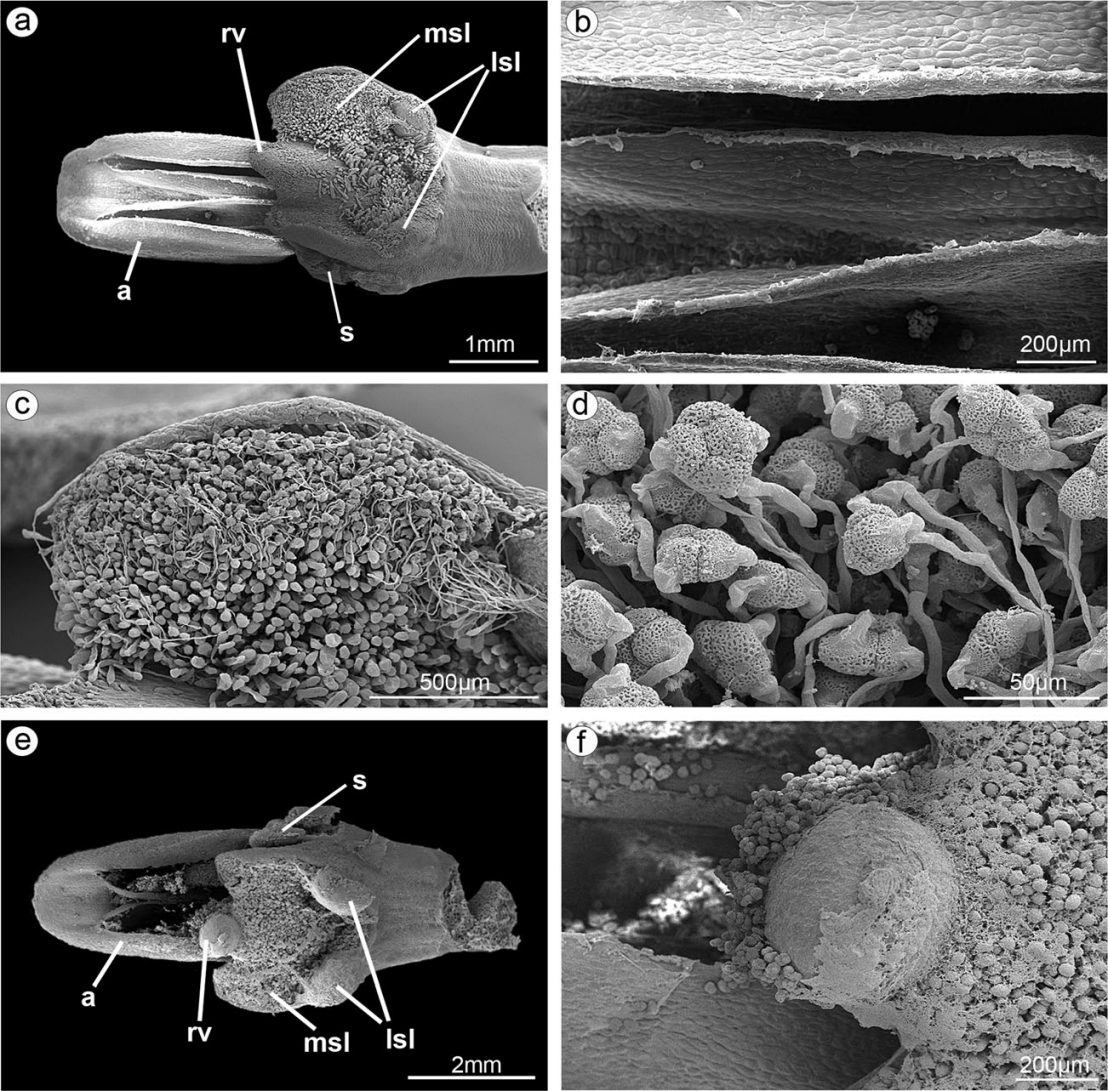
SEM illustrations of gynostemium from adult flowers at anthesis. **a** Empty anthers, pollen grains grouped in tetrads germinated on stigma. **b** Magnification of **a**, empty anthers. **c** Magnification of **a**, pollen grains germinated on median stigmatic lobe. **d** Magnification of **c**, germinating of pollen tetrads. **e** Pollen fallen out from anthers of this plant and adhering to the rostellum-viscidium. **f** Magnification of **e**, pollen grains from the anthers of this plant but germination of pollen grains not observed (*a* anther, *lsl* lateral stigmatic lobe, *msl* median stigmatic lobe, *rv* rostellum-viscidium, *s* staminodium)

The anthers in adult flowers at anthesis were found empty (Fig. 12a, b), and on stigma, the germinated pollen grains grouped in tetrads were visible (Fig. 12c, d). Pollen fallen out from anthers of this plant and adhering to the viscidium was found in the gynostemium (Fig. 12e, f). Nevertheless, germination of pollen grains was not observed.

## Discussion

The morphological, histochemical, and ultrastructural analysis of flowers collected at anthesis provides strong evidence to conclude that the whole surface of lip callus and abaxial side of isthmus are secretory. The distended cuticle on the abaxial side of epichile underneath callus is confusing and possibly also secretory. The secretive cells formed single-layered epidermis on callus, whereas on isthmus, the secretory activity was indicated in single-layered epidermis and several layers of underlying parenchyma, supplied by collateral vascular bundles. The collateral vascular bundles in nectaries are noted in about 12.6 % of angiosperms (Frei 1955), so most researchers claim that sugar and water constituents of nectar are provided via sieve tubes to nectariferous cells (Fahn 2000; Pacini et al. 2003; Vassilyev 2003; Barrera and Nobel 2004). The collateral bundles were also identified in *Hexisea imbricata*, which was interpreted that sugars transported via phloem elements were deposited as starch in the nectary cells (Stpiczyńska et al. 2005). The nectariferous cells on callus and isthmus were small, resembling meristematic cells in sizes (Durkee 1983), with large nucleus. Comparing the results of different buds’ sizes, it seems that the exudation at first appears on callus, at early stages, later on isthmus. It could be a strategy to prolong the emission of volatile substances and nectar, and this means to prolong luring pollinators. Moreover, the results from TEM also support this conclusion. In cells of both places (callus and isthmus), a large vacuole occupied the most area and the cytoplasm was parietal. During secretory process, the vacuoles expanded to form central vacuole, when the secretion had ceased (Schnepf 1973). The plastids noted in callus were without starch, whereas the isthmus’ cells contained partly hydrolyzed starch. The starch, source of sugars for nectar or source of energy for highly metabolic processes (Durkee 1983), was hydrolyzed during secretion process, which was commonly observed in plastids in other orchids (e.g., Pais and Figueiredo 1994; Stpiczyńska 1997; Stpiczyńska et al. 2005) and other plants (e.g., Nepi et al. 1996; Razem and Davis 1999; Vesprini et al. 1999). The plastids within the nectary cells of *H. imbricata*, when the nectar secretion was at the highest level, comprised both starch and numerous plastoglobuli (Stpiczyńska et al. 2005), the same as in nectary cells on isthmus whereas cells on callus possessed plastids with hydrolyzed starch, which means that the secretion was at lower level than on isthmus. Some plastids, noted in callus, had polymorphic shapes, which were often related to a starch reduction, the same as in *H. imbricata* (Stpiczyńska et al. 2005). During the depletion of starch in callus cells, the number of plastoglobuli within the plastids increased, and also lipid bodies appeared in the cytoplasm whereas, in isthmus cells, proplastids with phytoferritin were noted. Moreover, the expanded system of ER profiles was sometimes in connection with lipid bodies in callus. Lipids were frequently noted in the nectary cells of other orchids (Figueiredo and Pais 1992; Stpiczyńska 1997; Stpiczyńska and Matusiewicz 2001; Stpiczyńska et al. 2004; Paiva 2009). Lipid bodies present in the cytoplasm were regarded as physical equivalents of production of volatiles in osmophores (Swanson et al. 1980; Pridgeon and Stern 1983; Curry et al. 1988), also observed in *Anacamptis* (Kowalkowska et al. 2012). In cells of nectaries of *E. atropurpurea*, the cell wall ingrowths were observed but not reported in nectaries of *Limodorum abortivum*, as in *E. palustris*. It is explained by the nectar chemistry. In *E. palustris* (Percival 1961) and in *L. abortivum* (Pais and Figueiredo 1994), sucrose dominant nectar was present, whereas in *E. atropurpurea* was hexose rich (Pais and Figueiredo 1994). According to model proposed by Fahn (1979), the sugars (pre-nectar) are transported through the symplast of the secretory parenchyma (as in *Citrus*, *Vinca*, *Lonicera*) and then are loaded to ER or dictyosomes, and after fusion with plasmalemma, the nectar is released to the external surface. Further results (Pridgeon and Stern 1983; Stpiczyńska 1997; Stpiczyńska et al. 2005; Paiva 2009) confirm this model of secretion. In *E. palustris*, endoplasmic reticulum was in contact with plasmalemma, as observed previously in *Restrepia* (Pridgeon and Stern 1983). Moreover, the vesicles fusing with plasmalemma were frequently reported in secretory cells of callus and isthmus, as in other orchid species, e.g., in *Gymnadenia* (Stpiczyńska and Matusiewicz 2001), *Anacamptis* (Kowalkowska et al. 2012), and *Bulbophyllum* (Kowalkowska et al. 2014). The vesicles were sometimes surrounded by invaginations of the plasmalemma, which is also a way of granulocrine secretion, as in *Anacamptis* (Kowalkowska et al. 2012). Under the pressure of accumulated substances, the cuticle ruptures and nectar is releasing to the exterior (Durkee 1983; Curry et al. 1991). The other way to exude nectar is in diffusive manner through thin secretory cell walls (Fahn 1952). In the third option, nectar is exuded through modified stomata (Rachmilevitz and Fahn 1975; Durkee et al. 1981; Davis and Gunning 1992; Davies et al. 2005; Nepi 2007). In *E. palustris*, small vacuoles were sometimes encircled by dictyosomes, which may be involved in transport of substances, but generally at the secretory stage, dictyosomes are less abundant in cytoplasm (Pridgeon and Stern 1983).

The structure of sepals with stomata and large substomatal cavities led us to the conclusion that abaxial side of sepals may be involved in fragrance emission, similarly as in *Acianthera* (Melo et al. 2010), but further lines of research are necessary to prove this hypothesis. In parenchyma, numerous idioblasts with raphides possibly help to prevent herbivory (Prychid and Rudall 1999).

The observation of gynostemium with pollen adhering to the rostellum-viscidium gives the additional insight that the globular rostellum-viscidium is not a barrier and the self-pollination is possible. Well-developed rostellum is considered to be the physical barrier between the anthers and stigma, preventing self-pollination (Bonatti et al. 2006; Tałałaj and Brzosko 2008). In most self-fertilized orchids, it is observed that rostellum does not develop, not completely develops, or degenerates during anthesis (Catling 1990). The rostellum reduction lessens the physical barrier between the male and female floral parts, while decrease of viscidium depresses the possibility of taking the pollinia by pollinators (Claessens et al. 1998; Pedersen and Ehlers 2000; Ehlers et al. 2002). Such reductions allowed the pollinia to fall downward straight to the stigma, resulting in self-pollination. Nevertheless, even a well-developed rostellum is not an important barrier. Our results of *E. palustris* prove and give the SEM illustrations of the observations done by Tałałaj and Brzosko (2008). Pollen grains were fallen near the rostellum-viscidium trapped in the sticky glue. Tałałaj and Brzosko (2008) reported that during floral senescence, the viscidium dries and the barrier of rostellum-viscidium becomes weaker, allowing the pollen grains to be moved to the median stigmatic lobe. Futhermore, in *E. helleborine* var. *papillosa* and *E. helleborine* var. *sayekiana* (Suetsugu 2013), a well-developed rostellum is not the most important barrier. Rather, a poorly developed clinandrium seemed to be the major factor in self-pollination mechanism. At the end of anthesis during floral senescence, the pollinia became enlarged and less compressed and fallen onto the upper edges of the stigma. In these both varieties, self-pollination is a facultative autogamous strategy, occurring mostly at the end of anthesis. Also, in *Epipactis microphylla*, in an open basal flower, germinated pollen was exposed on the dorsal surface of the rostellum-viscidium (Bonatti et al. 2006). The self-pollination mechanism in *E. palustris* is quaint and needs SEM and LM (cross-sections) illustrations to fully confirm ecological experiments.
